# The clinical presentation, utilization, and outcome of individuals with sickle cell anaemia presenting to urban emergency department of a tertiary hospital in Tanzania

**DOI:** 10.1186/s12878-018-0122-3

**Published:** 2018-09-17

**Authors:** Hendry R. Sawe, Teri A. Reynolds, Juma A. Mfinanga, Michael S. Runyon, Brittany L. Murray, Lee A. Wallis, Julie Makani

**Affiliations:** 1 Emergency Medicine Department, Muhimbili University of Health and Allied Sciences, P.O. Box 54235, Dar es salaam, Tanzania; 2 Emergency Medicine Department, Muhimbili National Hospital, Dar es salaam, Tanzania; 3 Department of Emergency Medicine, University of California San Francisco, San Francisco, CA USA; 4 Department of Emergency Medicine, Carolinas Medical Center, Charlotte, NC USA; 5 Division of Pediatric Emergency Medicine, Emory University School of Medicine/Children’s Hospital of Atlanta, Atlanta, GA USA; 6 Division of Emergency Medicine, University of Cape Town, Cape Town City, South Africa; 7 Nuffield Department of Medicine, University of Oxford, Oxford, UK; 8 Department of Haematology and blood transfusion, Muhimbili University of Health and Allied Sciences, Dar es Salaam, Tanzania

**Keywords:** Sickle cell anaemia, Emergency department, Anaemia, Sub-Saharan Africa

## Abstract

**Background:**

Sickle cell anaemia (SCA) is prevalent in sub-Saharan Africa, with high risk of complications requiring emergency care. There is limited information about presentation of patients with SCA to hospitals for emergency care. We describe the clinical presentation, resource utilization, and outcomes of SCA patients presenting to the emergency department (ED) at Muhimbili National Hospital (MNH) in Dar es Salaam, Tanzania.

**Methods:**

This was a prospective cohort study of consecutive patients with SCA presenting to ED between December 2014 and July 2015. Informed consent was obtained from all patients or patients’ proxies prior to being enrolled in the study. A standardized case report form was used to record study information, including demographics, relevant clinical characteristics and overall patients outcomes. Categorical variables were compared with chi-square test or Fisher’s exact test; continuous variables were compared with two-sample t-test or Mann-Whitney U-test.

**Results:**

We enrolled 752 (2.7%) people with SCA from 28,322 patients who presented to the MNH-ED. The median age was 14 years (Interquartile range [IQR]: 6–23 years), and 395 (52.8%) were female. Pain 614 (81.6%), fever 289 (38.4%) were the most frequent presenting complaint. Patients with fever, hypoxia, altered mental status and bradycardia had statistically significant relative risk of mortality of 10.4, 153, 50 and 12.1 (*p* < 0.0001) respectively, compared to patients with normal vitals. Overall, 656 (87.2%) patients received Complete Blood Cell counts test, of these 342 (52.1%) had severe anaemia (haemoglobin < 7 g/dl), and a 30.3 (*p* = 0.02) relative risk of relative risk of mortality compare to patients with higher haemoglobin. Patients who had malaria, elevated renal function test and hypoglycemia, had relative risk of mortality of 22.9, 10.4 and 45.2 (*p* < 0.0001) respectively, compared to patient with normal values. Most 534 (71.0%) patients were hospitalized for in patients care, and the overall morality rate was 16 (2.1%).

**Conclusions:**

We described the clinical presentation, management, and outcomes of patients with SCA presenting to the largest public ED in Tanzania, as well as information on resource utilization. This information can inform development of treatment guidelines, clinical staff education, and clinical research aimed at optimizing care for SCA patients.

## Background

Sickle cell disease (SCD) is one of the most common genetic diseases in the world, with the highest prevalence in sub-Saharan Africa, the Caribbean, the Middle East and India [1]. The morbidity and mortality from Sickle Cell Anaemia (SCA) in developed countries has improved significantly over the last five decades, with studies showing median survival rates beyond the fifth decade of life [2]. In developing countries, however, SCA still causes significant morbidity and mortality with the greatest burden of the disease in sub-Saharan Africa. The World Health Organization (WHO) estimates over half of children born with SCA in sub-Saharan Africa will die before they reach adulthood [3]. Infectious diseases, such as malaria and pneumococcal disease (meningitis, pneumonia, and septicaemia) are thought to be the major cause of morbidity and mortality [4, 5].

Tanzania ranks fifth in the world for number of SCD births with an estimated prevalence of 6 [interquartile range (IQR) 1–13] per 1000 births, equivalent to 11,000 SCD births per year [4, 6]. A study conducted at a university teaching hospital in Tanzania reported a SCA mortality rate of 1.9 per 100 person years of observation (PYO), with the highest incidence of death occurring in the first five years of life [7]. Overall median survival in this study was 33 years. Of note, life expectancy of Tanzanian residents is 52 years. Similar to other developing countries, morbidity and mortality due to SCA in Tanzania is highly influenced by comorbid conditions, such as infections (particularly malaria), anaemia, and poor nutrition [4].

Emergency department (ED) presentations of SCA complications are well characterized in developed countries, with the majority of these patients presenting with pain crisis [8–12]. Most of these patients are treated with aggressive pain management and fluid therapy and discharged home, with less than one-third admitted to the hospital. In 2010, Muhimbili National Hospital (MNH), which is the main University Teaching Hospital, opened the first full capacity, high-volume ED in Tanzania. This ED has provided an opportunity for early stabilization and resuscitation of acutely ill patients, including those with SCA. The study on mortality in SCA in Africa published in 2011 was conducted at the same hospital, at that time had no full capacity ED [7]. This is critical as little is known about the acute or emergent manifestations, management, and outcomes of SCA complications in Tanzania. The primary aim of this investigation is to describe the clinical presentation, ED management, and hospital outcomes of individuals known or suspected to have sickle cell disease and presenting to the MNH ED.

## Methods

### Study design and setting

This was a prospective observational study of consecutive patients presenting to the MNH ED in Dar es Salaam, Tanzania, from 1 December 2014 to 31 July 2015. MNH has a bed capacity of 1500 and serves as the top referral hospital in Tanzania. The ED was established in 2010 via a partnership between the Ministry of Health and Social Welfare, and Abbott Fund Tanzania. The ED is the first full capacity public ED in Tanzania, and the training site for the only emergency medicine (EM) residency program in the country. The department is staffed 24 h, seven days a week by locally trained specialist emergency physicians, who oversee the care of patients and training of interns, registrars, and EM residents. The ED sees an average of 45,000 patients annually, with an admission rate of around 65%. The top disease categories in all age groups are trauma, infectious disease, and mental health [13]. MNH is one of the largest SCD treatment and research centre in Tanzania. The diagnosis of SCA at MNH is normally confirmed by the haemoglobin electrophoresis, after initial sickling test or clinical suspicion at most of the referring hospitals. Patients confirmed to have SCA are enrolled into a Sickle Cell clinic, provided with dedicated medical record number and special follow up card. In this study, all patients with confirmed evidence of SCA presenting with acute illness at the ED were eligible for enrolment.

### Study protocol

Screening and enrolment was completed by a research assistant and the study investigator (HS). A structured data sheet was used to record study information, including demographics, pre-referral information, chief complaints, initial vital signs, history and physical examination findings, laboratory results, treatment delivered, and final ED diagnoses and disposition. All diagnostic, treatment, and disposition decisions were made at the discretion of the treating physician. The research assistant followed up on all enrolled patients throughout the duration of their hospital study and recorded lengths of stay in hospital and intensive care unit (ICU) or high acuity ward, as well as final hospital diagnosis and clinical outcome (discharge or death) for each patient.

### Data analysis

Information collected from the handwritten data sheets were transferred into an Excel spreadsheet (Microsoft Corporation, Redmond, WA, USA), verified, and checked for any errors and outliers. Data were subsequently imported into StatsDirect (version 3.0.167, StatsDirect Ltd., Cheshire, UK) for analysis. Categorical variables were summarized as frequencies and percentages, and continuous variables as means and standard deviations (SD) or medians and interquartile ranges (IQR), depending on data distribution. Normality was assessed using the Shapiro-Wilk test. Ninety-five percent confidence intervals (CI) are presented where appropriate, and were calculated by the Clopper-Pearson (exact) method. Categorical variables were compared with chi-square test or Fisher’s exact test; continuous variables were compared with two-sample t-test or Mann-Whitney U-test. Two-tailed *p*-values of < 0.05 were considered statistically significant.

## Results

We enrolled 752 (2.7%) people with SCA from 28,322 patients who presented to the MNH ED from 1 December 2014 to 31 July 2015. The median age of enrolled patients was 14 years (Interquartile range (IQR) of 6–23 years), with 19.7% younger than age 5, and 52.9% were female. A total of 299 (40.2%) patients were referred from peripheral hospitals, the median length of admission at these peripheral facilities prior to referral was 2 days (IQR 1.3 days) Table 1.

**Table 1:** Patient demographics

Demographics	Number	Percentage (%)
Sex	*N* = 747	
Female	395	52.9
Male	352	47.1
Age Group	*N* = 741	
1 month–< 5 years	146	19.7
5 years–< 18 years	304	41.0
≥ 18 years	291	39.3
Referral Status	*N* = 744	
Self referral	445	59.8
Referred	299	40.2

### Patients’ baseline variables and presenting complaints

Tachypnea 336 (44.7%) was the most frequent abnormal vital sign among enrolled patients, while bradycardia 14 (1.9%) was the least frequent abnormal sign. All the abnormality are based on age-appropriate vital signs [14]. Patients who presented fever 289 (17.4%), hypoxia 67 (8.9%), altered mental status 59 (7.8%) and bradycardia 14 (1.9%) had a statistically significant higher relative risk of death compared with those without bradycardia. Pain 614 (81.6%) and urinary symptoms 6 (0.8%) were most and least frequent presenting complaints respectively Table 2.

**Table 2:** Patients’ baseline variables and presenting complaints

	Overall	Died	Survived	Relative risk	*P*-value
	*N* = 752	*N* = 16	*N* = 736		
Clinical characteristics	n (%)	n (%)	n (%)	RR (95%CI)	
Tachypnea ^b^	336 (44.7)	10 (62.5)	326 (44.3)	2.1 (0.8–5.7)	0.16
Tachycardia^b^	186 (24.7)	5 (31.3)	181 (24.6)	1.4 (0.5–3.9)	0.5
Febrile (*T* > 37.5 °C)^c^	131 (17.4)	11 (68.8)	120 (16.3)	10.4 (3.7–29.5)	< 0.0001
SpO_2_^a^ < 95%	67 (8.9)	15 (93.8)	52 (7.1)	153 (20–1143)	< 0.0001
Altered mental status	59 (7.8)	13 (81.3)	46 (6.3)	50 (15–174)	< 0.0001
Bradycardia^b^	14 (1.9)	3 (18.8)	11 (1.5)	12.1 (3.9–38.0)	< 0.0001
Pain	614 (81.6)	12 (75.0)	602 (81.8)	0.7 (0.2–2.1)	0.5
Fever	289 (38.4)	10 (62.5)	279 (37.9)	2.7 (1.0–7.3)	0.06
Abdominal Symptoms	159 (21.1)	2 (12.5)	157 (21.3)	0.5 (0.1–2.3)	0.4
Respiratory Symptoms	156 (20.7)	11 (68.8)	145 (19.7)	8.4 (3.0–23.8)	0.0001
Cardiovascular Symptoms	83 (11.0)	2 (12.5)	81 (11.0)	1.2 (0.3–5.0)	0.9
Jaundice	80 (10.6)	1 (6.3)	79 (10.7)	0.6 (0.1–4.2)	0.6
Body Swelling	54 (7.2)	2 (12.5)	52 (7.1)	1.9 (0.4–7.9)	0.4
Neurological Symptoms	45 (6.0)	6 (37.5)	39 (5.3)	9.4 (3.6–24.8)	< 0.0001
Long Lasting Erection	20 (2.7)	1 (6.3)	19 (2.6)	2.4 (0.3–17.6)	0.4
Urinary Symptoms	6 (0.8)	1 (6.3)	5 (0.7)	8.3 (1.3–53.1)	0.03

^a^*SpO*_*2*_ Saturation of oxygen in peripheral capillary ^b^Age-adjusted variables ^c^ Measurements were all axillary

### Investigations ordered in the ED

Nearly all patients 744 (99%) had at least one laboratory test done while receiving ED care. Complete blood cell counts were ordered for 656 (87.2%) patients. Of these 346 (52.7%) had elevated white blood counts (> 11 K/uL) and 342 (52.1%) had severe anaemia (Hb < 7 g/dL) of which 166 (25.3%) had (Hb < 5 g/dL). Of the 415 patients tested for malaria, 48 (11.6%) were positive. The relative risk of death among those with severe anaemia, malaria test positive, elevated renal function test and hypoglycaemia was 30.3, 22.9,10.4 and 45.2 respectively. X-ray of the chest was ordered in 85 (11.3%) of patients, and the relative risk of death among those with an abnormal chest x-ray was 4.0 (Table 3).

**Table 3:** Investigations ordered in the ED

	Overall	Died	Survived	Relative risk	*P*-value
	n/N (%)	n/N (%)	n/N (%)	RR (95%CI)	
Laboratory Tests					
WBC ^**Ω**^ (> 11 K/uL)	346/656 (52.7)	11/16 (68.8)	335/640 (52.3)	2.0 (0.7–5.6)	0.2
Haemoglobin (< 7 g/dL)	342/656 (52.1)	16/16 (100)	326/640 (50.9)	30.3 (1.8–503)	0.02
Abnormal urine results ^**δ**^	11/63 (17.5)	1/16 (6.3)	10/47 (21.3)	0.3 (0.04–2.11)	0.2
Malaria test positive	48/415 (11.6)	12/16 (75)	36/399 (9.0)	22.9 (7.7–68)	< 0.0001
Elevated RFT^**β**^	24/219 (11.0)	9/16 (56.3)	15/203 (7.4)	10.4 (4.2–25.5)	< 0.0001
Low RBG^**η**^ (< 3 mmol/L)	39/627 (6.2)	12/16 (75)	27/611 (4.4)	45.2 (15.3–133.8)	< 0.0001
Imaging Tests					
Abnormal chest x-ray ^**α**^	30/85 (35.3)	11/16 (68.8)	19/69 (27.5)	4.0 (1.5–10.5)	0.004
Abnormal brain CT scan ^**ρ**^	7/26 (26.9)	5/16 (31.3)	2/10 (20)	1.2 (0.67–2.2)	0.5

^Ω^ WBC-White Blood Cell

^δ^ Presence of blood in urine, leukocytes, nitrites, albumin, or glucose

^α^ Signs of infection or stroke

^β^ RFT-Renal function test

^η^ RBG-Random Blood Glucose

^α^ Pneumonic changes

^ρ^ Signs of stroke

### Final ED diagnosis

The top three ED diagnoses were painful crisis (*n* = 472; 62.8%), malaria (*n* = 176; 23.4%), and severe anaemia (*n* = 117; 15.6%) (Table 4).

**Table 4:** Final ED diagnosis

Final ED Diagnosis	*N* = 752	% (95% CI)
Painful crisis	472	62.8 (59.3–66.2)
Malaria	176	23.4 (20.5–26.6)
Severe anaemia	117	15.6 (13.2–18.3)
Pneumonia	105	14.0 (11.7–16.6)
Acute chest syndrome	49	6.5 (5.0–8.5)
Hypoglycaemia	42	5.6 (4.2–7.5)
Urinary tract infection	41	5.5 (4.0–7.3)
Acute kidney injury	27	3.6 (2.5–5.2)
Priapism	18	2.4 (1.5–3.8)
Cerebral vascular accident	16	2.1 (1.3–3.4)

### ED treatment and interventions

In the ED, intravenous fluid bolus and intravenous dextrose were given to 370 (49.2%) and 129 (17.2%) of enrolled patients, respectively. A total of 489 patients (65.0%) received analgesics for pain, with 350 (71.6%) receiving opioid analgesics. Antimalarial were administered in the ED to 123 (16.4%) patients, while 220 (29.3%) received antibiotics, the majority of whom (89.2%) received intravenous ceftriaxone. Seventy-two patients (9.6%) received blood products (fresh whole blood, packed red cells, or fresh frozen plasma), with the majority (91.2%) receiving fresh whole blood.

### Patients’ disposition and hospital outcomes

Of the 752 SCA patients seen in the ED, 534 (71%) were hospitalized for inpatient care, while five patients (0.7%) died in the ED. The median length of stay in hospital was 3 days (Interquartile range (IQR): 1–5) days. The overall morality (ED plus inpatient) was 16 (2.1%). Overall, 8 (50%) of deaths’ occurred within 24 h of ED presentation (Table 5).

**Table 5:** Patients’ disposition and hospital outcomes

Disposition	*N* = 752	% (95% CI)
Admitted	534	71.0 (67.6–74.2)
Discharged from ED	213	28.3 (25.1–31.7)
Died in ED	5	0.7 (0.3–1.5)
Death within 24-h of ED presentation ^ϖ^	8	1.1 (0.5–2.1)
Inpatient mortality ^φ^	11	2.1 (1.2–3.7)
Overall mortality	16	2.1 (1.3–3.4)
	Median	IQR
Median length of hospital stay	3 days	1–5 days

ϖ Includes ED and inpatient deaths

φ Includes only in patients’ deaths (denominator: *N* = 534) those who were admitted

## Discussion

The opening of a full capacity ED at MNH provided a unique opportunity for rapid assessment and early stabilization of acutely ill individuals, including those with SCA. Our study reports on the clinical profile and management of acutely ill individuals with SCA presenting to an urban ED in Tanzania. This information on ED access and resource utilization can be useful in developing local and countrywide strategies to improve access, treatment guidelines, and health outcomes among individuals with SCA.

In our study, the prevalence of SCA among acutely ill patients presenting ED was found to be 2.7%, and most of the patients were self-referral, highlighting the role of ED as a major mode of healthcare utilization in this patient population in Tanzania. Most of our patients were children below eighteen years, an observation consistent with previously published literature in the same settings [7].

Pain was the most common presenting complaints among patients presenting to ED. Vaso-occlusive painful crisis phenomena is a well documented reason for ED visit among sickle cell patients in different settings [7, 8], and it has been shown to be a potential marker of serious illness, which may be associated with increased morbidity and mortality [7, 15]. Respiratory compromise, denoted by tachypnea and hypoxia (oxygen saturation < 95%), was the most common physical examination finding at presentation. In this population, the most common reason for respiratory compromise was chest infection (pneumonia), followed by acute chest syndrome. Infection is the leading cause of preventable morbidity and mortality in patients with SCA, and previous studies reported mortality as high as 38% in the USA, in absence of aggressive treatment [16, 17]. Standardized treatment and prevention (i.e. prophylactic penicillin and vaccination against pneumococcal infections) have significantly improved the outcome and quality of life [18]; however, these interventions are not routinely available to our patient population as they are not part of standard care [19]. When present, bradycardia, altered mental status, and hypoxia were each associated with a relative risk of death of greater than 10 in our study population compared to when these features were absent.

In addition to physical examinations, most of these patients received laboratory and imaging tests. More than three-quarters of patients had a complete blood cell count as part of their ED care, more than half of who had severe anaemia. All patients who died had severe anaemia at presentation. Severe anaemia was associated with a significantly higher relative risk of death when compared with higher haemoglobin levels. These findings are similar to previous literature in similar settings, which have shown low haemoglobin to be an independent predictor of mortality in patients with SCA [16, 20]. Severe anaemia is reported to negatively impact outcome and quality of life in SCA patients [20]. In our patient cohort, only 21% of patients with severe anaemia received blood transfusion in the ED. The current local guideline for management of severe anaemia in patients with SCA recommends blood transfusion to all SCA patients with haemoglobin (Hb) less than 5 g/dL, regardless of presenting complaint, and to those with Hb < 7 g/dL if symptomatic [21]. In this study population, 25.3% of patients had haemoglobin less than 5 g/dL, but overall more than half of patients who should receive blood products as part of their ED care did not receive it. Several factors may impact availability, access, and transfusion practices in our ED, including lack of enough eligible donors, lack of testing and storage equipment, lack of recognition and late presentation [22, 23]. We are unable to determine the exact reasons for the low rate of transfusion in our study. Thus, we recommend further study to address what appears to be a significant opportunity for improvement in emergency care of acute SCA cases. The findings of hypoglycaemia, positive malaria test, abnormal renal function tests and abnormal chest x-ray findings were all associated with a significantly higher relative risk of death compared to patients with normal results. All of these abnormal findings are easily manageable at MNH, and at most of the basic facilities within Tanzania, hence further studies should focus on addressing the challenges associated with care and factors associated with mortality within these conditions so as to ensure death from these conditions are prevented.

Painful crisis, malaria and severe anaemia were the top three ED providers’ diagnosis. Malaria was diagnosed in 176 patients by ED providers, while only 48 of patients tested positive, this might indicate an over diagnosis by ED providers, however we were unable to detect whether the providers used syndrome approach to treatment of malaria which calls for use of antimalarial for suspicious cases regardless of the malaria test results [24].

Over two-thirds of patients were admitted for inpatient care after initial stabilization in the ED. This is a much higher admission rate than reported in many developed countries [8, 10, 12], but similar to the overall ED disposition patterns in our setting [13]. The overall mortality in our study population was 2.1%, with 50% of the deaths occurring within the first 24 h of presentation to the ED. Our results show that severe anaemia, infection (malaria and bacterial infection), and hypoxia are associated with increase in mortality in our study population. These contributors should receive focused attention in the design of ED care protocols for SCA and further studies.

### Limitations

The generalizability of our results may be limited due to the single-centre nature of our study. The ED at MNH is the entry point to the largest tertiary referral hospital in Tanzania, and receives acutely ill patients from all over the country. Therefore, the sample seen at the MNH ED might be different than that seen at the district hospitals and health centres. The exclusion of undiagnosed SCD in our study might have under-estimated the overall proportion of SCD in our study population. In addition, the assessment of factors associated with mortality was limited by the number of deaths in our study population.

## Conclusions

We have provided a description of the clinical presentation, management, and outcomes of patients with SCA presenting to the largest public emergency department in a tertiary referral hospital in Tanzania. These data will inform development of strategies to provide education for clinical staff, treatment guidelines, and clinical research aimed at optimizing care of the SCA patient population.

## Abbreviations

CBC: Complete Blood Count
CRF: Case Report Form
ED: Emergency Department
MNH: Muhimbili National Hospital
MUHAS: Muhimbili University of Health and Allied Sciences
WHO: World Health Organization

## References

[1] CDC - Sickle Cell Disease, Data and Statistics - NCBDDD [Internet]. [cited 2013 Mar 13]. Available from: http://www.cdc.gov/ncbddd/sicklecell/data.html.

[2] Platt OS, Brambilla DJ, Rosse WF, Milner PF, Castro O, Steinberg MH (1994). Mortality in sickle cell disease – life expectancy and risk factors for early death. N Engl J Med.

[3] Weatherall DJ (2010). The inherited diseases of hemoglobin are an emerging global health burden. Blood.

[4] Fleming AF (1989). The presentation, management and prevention of crisis in sickle cell disease in Africa. Blood Rev.

[5] Oniyangi O, Omari AA. Malaria chemoprophylaxis in sickle cell disease. In: Cochrane Database of Systematic Reviews [Internet]. John Wiley & Sons, Ltd; 1996 [cited 2013 Mar 15]. Available from: https://www.ncbi.nlm.nih.gov/pubmed/17054173.

[6] Piel FB, Patil AP, Howes RE, Nyangiri OA, Gething PW, Dewi M (2013). Global epidemiology of sickle haemoglobin in neonates: a contemporary geostatistical model-based map and population estimates. Lancet.

[7] Makani J, Cox SE, Soka D, Komba AN, Oruo J, Mwamtemi H (2011). Mortality in sickle cell anemia in Africa: a prospective cohort study in Tanzania. PLoS One.

[8] Tanabe P, Myers R, Zosel A, Brice J, Ansari AH, Evans J (2007). Emergency department management of acute pain episodes in sickle cell disease. Acad Emerg Med.

[9] Tanabe P, Hafner JW, Martinovich Z, Artz N (2012). Adult emergency department patients with sickle cell pain crisis: results from a quality improvement learning collaborative model to improve analgesic management. Acad Emerg Med.

[10] Yusuf HR, Atrash HK, Grosse SD, Parker CS, Grant AM (2010). Emergency department visits made by patients with sickle cell disease: a descriptive study, 1999-2007. Am J Prev Med.

[11] Gray A, Anionwu EN, Davies SC, Brozovic M (1991). Patterns of mortality in sickle cell disease in the United Kingdom. J Clin Pathol.

[12] Reddin CDRC, Cerrentano E, Tanabe P (2011). Sickle cell disease management in the emergency department: what every emergency nurse should know. J Emerg Nurs.

[13] Reynolds T, Sawe HR, Lobue N, Mwafongo V (2012). Most frequent adult and pediatric diagnoses among 60,000 patients seen in a new urban emergency Department in Dar Es Salaam, Tanzania. Ann Emerg Med.

[14] Fleming S, Thompson M, Stevens R, Heneghan C, Plüddemann A, Maconochie I (2011). Normal ranges of heart rate and respiratory rate in children from birth to 18 years: a systematic review of observational studies. Lancet.

[15] Heimlich JB, Chipoka G, Kamthunzi P, Krysiak R, Majawa Y, Mafunga P (2016). Establishing sickle cell diagnostics and characterizing a paediatric sickle cell disease cohort in Malawi. Br J Haematol.

[16] Autino B, Noris A, Russo R, Castelli F. Epidemiology of Malaria in Endemic Areas. Mediterr J Hematol Infect Dis [Internet]. 2012 [cited 2016 Aug 20];4(1). Available from: http://www.ncbi.nlm.nih.gov/pmc/articles/PMC3499992/. 10.4084/MJHID.2012.060 PMC3499992 23170189

[17] Booth C, Inusa B, Obaro SK (2010). Infection in sickle cell disease: a review. Int J Infect Dis.

[18] Luzzatto L. Sickle Cell Anaemia and Malaria. Mediterr J Hematol Infect Dis [Internet]. 2012 [cited 2016 Aug 20];4(1). Available from: http://www.ncbi.nlm.nih.gov/pmc/articles/PMC3499995/. 10.4084/MJHID.2012.065 PMC3499995 23170194

[19] Leikin SL, Gallagher D, Kinney TR, Sloane D, Klug P, Rida W (1989). Mortality in children and adolescents with sickle cell disease. Cooperative study of sickle cell disease. Pediatrics.

[20] Cox SE, Makani J, Fulford AJ, Komba AN, Soka D, Williams TN (2011). Nutritional status, hospitalization and mortality among patients with sickle cell anemia in Tanzania. Haematologica.

[21] Haematology and Blood Transfusion M. Management of Sickle Cell Disease [Internet]. Department of Internal Medicine, Muhimbili National Hospital, Dar es salaam-Tanzania; 2014. Available from: https://www.h3abionet.org/muhas.

[22] Vos Jennechien, Gumodoka Balthazar, van Asten Henri A., Berege Zachariah A., Dolmans Wil M., Borgdorff Martien W. (1994). Changes in blood transfusion practices after the introduction of consensus guidelines in Mwanza region, Tanzania. AIDS.

[23] Gumodoka B, Vos J, Kigadye FC, van Asten H, Dolmans WM, Borgdorff MW (1993). Blood transfusion practices in Mwanza region, Tanzania. Bugando Medical Centre. AIDS.

[24] Information NC for B, Pike USNL of M 8600 R, MD B, Usa 20894. TREATMENT OF SEVERE MALARIA [Internet]. World Health Organization; 2015 [cited 2017 Sep 24]. Available from: https://www.ncbi.nlm.nih.gov/books/NBK294445/.

